# Computational Study of Cresyl Violet Covalently Attached to the Silane Coupling Agents: Application to TiO_2_-Based Photocatalysts and Dye-Sensitized Solar Cells

**DOI:** 10.3390/nano10101958

**Published:** 2020-10-01

**Authors:** Tatsuya Takeshita

**Affiliations:** Department of Applied Chemistry and Food Science, Fukui University of Technology, 3-6-1 Gakuen, Fukui 910-8505, Japan; takeshita@fukui-ut.ac.jp

**Keywords:** cresyl violet, silane coupling agents, titanium dioxide, photocatalysts, dye-sensitized solar cell, DFT and TD-TDF calculation

## Abstract

The covalent attachment of photosensitizing dyes to TiO_2_ using silane coupling agents (SCAs) is a promising strategy for enhancing the photocatalytic activity of TiO_2_-based photocatalysts and the photovoltaic conversion of dye-sensitized solar cells (DSSCs). This approach can control the geometry and orientation of the photosensitizing dye on the TiO_2_ surface. In this study, a density functional theory (DFT) and time-dependent DFT (TD-DFT) investigation was carried out on cresyl violet (CV) covalently attached to SCAs with a terminal oxirane group (OTES–Cn) to reveal the influence of OTES–Cn on the geometry of the photosensitizing dyes. The potential of CV covalently attached to OTES–Cn (CV–OTES–Cn) to act as a photosensitizing dye was also analyzed. The hydroxyl group formed by the epoxy-opening reaction between CV and OTES–Cn strongly influenced the geometry of CV–OTES–Cn, which was attributed to a CH–O interaction. Additionally, TD-DFT, frontier molecular orbital and molecular electrostatic potential calculations revealed that CV–OTES–Cn has excellent optical properties and electron injection ability. In particular, the characteristics of the unbent conformation of CV–OTES–Cn are expected to contribute significantly to the photocurrent in TiO_2_-based photocatalysts and DSSCs. These findings enhance the understanding of the covalent attachment strategy using SCAs and contribute to improving TiO_2_-based photocatalysts and DSSCs.

## 1. Introduction

Photoredox catalysts play an essential role in various fields, including environmental remediation, solar energy conversion and organic photochemistry [1,2,3]. Titanium dioxide (TiO_2_) is a typical photoredox catalyst with strong oxidation and reduction abilities [4]. Generally, TiO_2_ exhibits photocatalytic behavior under UV light with a photon energy corresponding to the bandgap between its conduction band (CB) and valence band levels. Hence, modification is required to enhance its photocatalytic activity and absorb visible light, as the UV region represents only ~4% of the solar spectrum [5,6].

Surface and interface modifications are useful strategies for fabricating visible-light-responsive TiO_2_. In particular, the chemical adsorption of organic dyes onto the surface of TiO_2_ is a simple method to impart it with a visible light response; the photosensitization occurs via electron injection from the adsorbed organic dyes into the CB of TiO_2_. Using this strategy, dye-sensitized solar cells (DSSCs), which are one of the main applications of TiO_2_, with a photovoltaic conversion efficiency (PCE) of 14.3% have been achieved [2].

In addition to the photosensitizing dye’s visible light response, the dye’s geometry on the TiO_2_ surface also strongly affects the electron injection efficiency. Improving the photosensitizing dye orientation can reduce unwanted electron transfer between dye molecules, which occurs due to aggregation of the dye and back-electron transfer between the oxidized dye and the electrons injected into TiO_2_ [7,8].

In the design and synthesis of photosensitizing dyes, most investigations of methods to improve the dye’s orientation on the TiO_2_ surface have focused on attaching anchor groups to the dye [7,8,9]. However, most photosensitizing dyes have complex molecular structures designed to improve their optical properties (extended π-conjugation, push–pull structures, etc.) [2,10,11,12]. Adding anchor groups to these dyes to improve their orientation requires multiple synthetic steps and can be expensive.

Silane coupling agents (SCAs) consist of an alkyl alkoxysilane moiety and two or more different functional groups that enable the SCAs to covalently attach to both inorganic and organic materials [13,14]. Because SCAs can anchor photosensitizing dyes to the TiO_2_ surface, they represent an easy and low-cost covalent-attachment strategy that can be used to enhance TiO_2_-based photocatalysis and the PCEs of DSSCs [1,6,15]. Recently, Lu et al. reported improvement of the visible photocatalytic activity of TiO_2_ using glycidoxypropyltrimethoxy silane and zinc (II) tetra-[α-(*p*-amino)benzyloxyl]phthalocyanine [1]. This strategy has also been applied to DSSCs [16,17]. Zamborini et al. reported that the stability of a DSSC was improved by using 3-aminopropyltriethoxysilane [16].

In addition to covalently linking inorganic and organic materials, SCAs can also form self-assembled monolayers (SAMs), attributed to the formation of a siloxane network [13,14]. The aligned orientation of these SAMs could be exploited to control a photosensitizing dye’s geometry and orientation on a TiO_2_ surface. Thus, understanding the geometry and characteristics of photosensitizing dyes covalently attached to SCAs is essential to enhance further the photocatalytic activity of TiO_2_-based photocatalysts and the PCEs of DSSCs.

**Figure 1:**
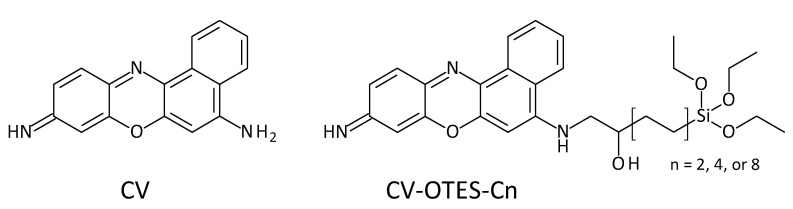
Chemical structures of cresyl violet (9-iminobenzo[a]phenoxazin-5-amine; CV) and CV–OTES–Cn. For the chemical structures of OTES–Cn, see Figure S1.

This article reports a density functional theory (DFT) and time-dependent DFT (TD-DFT) study of cresyl violet (CV, Figure 1) covalently attached to SCAs with a terminal oxirane group (OTES–Cn, Figure S1; Cn denotes the number of carbons in the alkyl chain, *n* = 2, 4 or 8). DFT and TD-DFT are powerful tools for understanding the geometry and characteristics of molecules. CV was chosen as the photosensitizing dye in this study based on its excellent optical properties. It contains an amino group that can form a covalent bond with an oxirane via an epoxy-ring-opening reaction between the amino and epoxy groups. Additionally, phenoxazine-based dyes are known to show high performance in DSSCs [18,19,20]. To the best of the author’s knowledge, this is the first investigation of the combination of CV and OTES–Cn. This investigation will reveal both the influence of OTES–Cn on the geometry of photosensitizing dyes and CV’s potential to attach to OTES–Cn covalently (CV–OTES–Cn) and act as a photosensitizing dye.

## 2. Computational Methods

Geometry optimization, frontier molecular orbital (FMO), UV-visible absorption spectrum simulation, frequency analysis and molecular electrostatic potential (MEP) calculations were carried out using the Gaussian 09W package (Gaussian, Inc., Wallingford, CT, USA). The density of state (DOS) spectra were obtained using GaussSum (3.0.2) [21]. The geometries of all dyes were optimized in the gas-phase without symmetry constraints. The B3LYP level of theory with the 6-31G* basis set (B3LYP/6-31G* level) was employed for geometry optimizations, FMO calculations, frequency analyses and MEP calculations, except in the case of protonated CV (CV^+^, see below). In calculations involving TiO_2_, the LanL2DZ basis set was assigned to the titanium atoms [22]. TD-DFT calculations (UV-visible absorption spectrum simulations) were performed at the B3LYP/6-31+G* level of theory. Geometry optimization and simulation of the UV-visible absorption spectra of CV^+^ were carried out using the B3LYP/6-31+G** and SVWN/6–31+G** levels of theory. For the MEP calculation, the van der Waals radius of the titanium atoms was specified as 2.2 Å [23,24]. The conductor-like polarized continuum model (CPCM) using ethanol (EtOH) was employed for all calculations determining the solvent effect, except the geometry optimizations.

## 3. Results and Discussion

### 3.1. Characteristics of CV

CV is a basic dye and coexists with counterions such as acetate and perchlorate. Hence, CV has multiple chemical structures. In this study, neutral CV (9-iminobenzo[a]phenoxazin-5-amine), its isomer (5-iminobenzo[a]phenoxazin-9-amine) and protonated CV are denoted as CV_0_, CV_iso_ and CV^+^, respectively. The optimized structures of CV_0_, CV_iso_ and CV^+^ in the gas phase are illustrated in Figure S2. For CV^+^, structures with the positive charge at various locations (NH_2_^+^, O^+^ and N^+^) can be considered. However, these structures are not distinguished in the present work because this charge is delocalized over the entire molecule in DFT calculations [25]. Therefore, CV^+^ was optimized as a structure with two amino groups and a positive charge distributed over the entire molecule.

**Figure 2:**
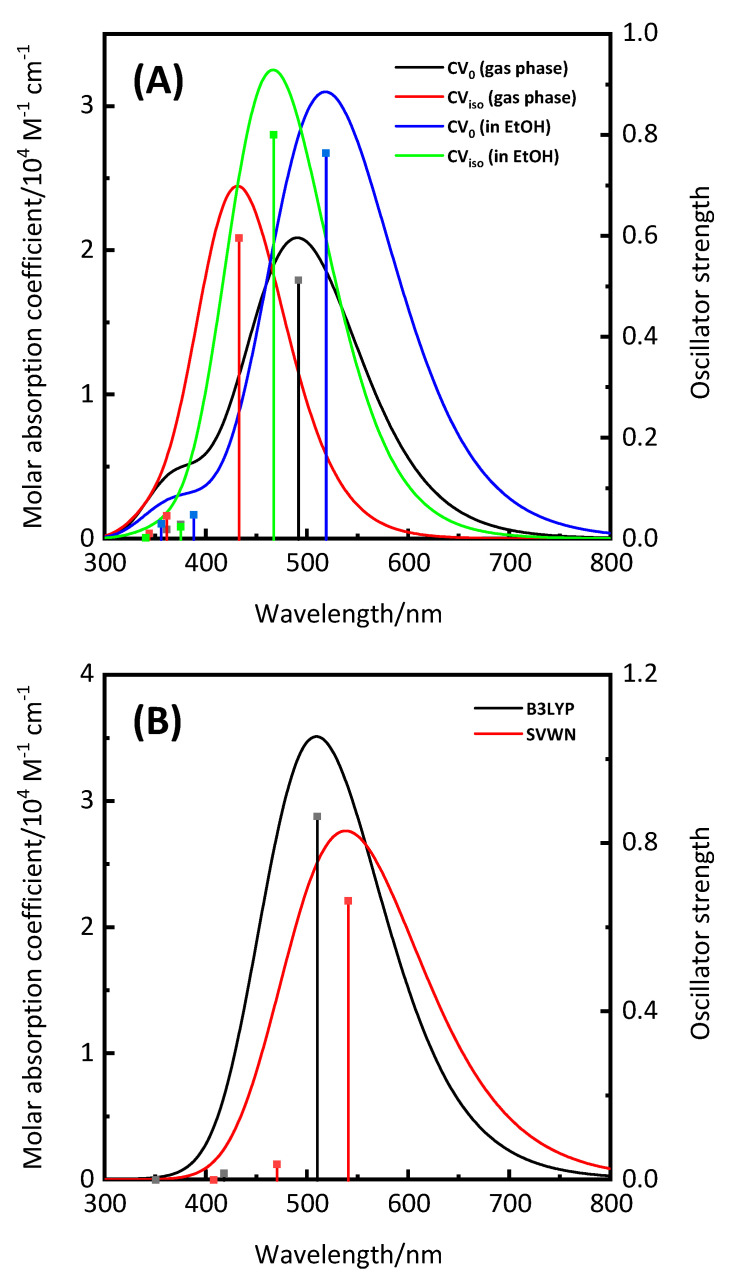
(**A**) Simulated UV-visible absorption spectra of CV_0_ and CV_iso_ in the gas phase and/or using CPCM in EtOH, calculated at the TD-B3LYP/6-31+G* level of theory; (**B**) simulated UV-visible absorption spectra of CV^+^ using CPCM in EtOH, calculated at the TD-B3LYP/6-31+G** (black line) and TD-SVWN/6-31+G** (red line) levels of theory, respectively. Vertical lines indicate the calculated oscillator strengths.

Next, the UV-visible absorption spectra of CV in the gas phase were simulated (Figure 2A, black and red lines), as the photosensitizing dye’s optical properties are among the most important factors in determining the photocatalytic activity of TiO_2_-based photocatalysts and the PCEs of DSSCs [12]. The maximum absorption peaks (*λ*_max_) of CV_0_ and CV_iso_ were observed at 491 and 432 nm, respectively (Table 1) [26]. In addition, the oscillator strengths (*f*) at *λ*_max_ were calculated to be 0.513 and 0.5968, respectively. Good light-harvesting efficiency (LHE) is important for obtaining high photocurrent in TiO_2_-based photocatalysts and DSSCs; this parameter can be calculated from the *f* values using the following equation [10,11,27]:

LHE = 1 − 10^−*f*^ (1)

The LHEs of CV_0_ and CV_iso_ were calculated to be 0.69 and 0.75, respectively, using Equation (1). It was expected that the solvent effect would result in a shift in the UV-visible absorption spectra and changes in the LHE values. The simulated UV-visible absorption spectra of CV_0_ and CV_iso_ using CPCM in EtOH showed a red-shift, and their LHE values increased (Figure 2A, blue and green lines). These results suggested that CV_0_ and CV_iso_ have excellent optical properties as photosensitizing dyes.

Like CV_0_ and CV_iso_, the UV-visible absorption spectrum of CV^+^ was simulated using CPCM in EtOH (Figure 2B). The *λ*_max_ of CV^+^ was observed at 510 nm when the B3LYP/6-31+G** level of theory was used. High concentrations of CV^+^ in aqueous solutions have been reported to exhibit a dimeric band at ~550 nm, which is attributed to H-aggregated species [28]. Additionally, the absorption peak of CV^+^ on a Langmuir–Blodgett film is observed at ~525 nm [29]. On the other hand, the monomeric band of CV^+^ is known to have a *λ*_max_ of ~585 nm [25,28,29,30]. This band means that the energy of the charge–transfer state of CV^+^ was overestimated in the TD-DFT calculation at the B3LYP/6-31+G** level of theory. Thus, other calculation methods were investigated, and the SVWN method was found to show relatively good agreement (*λ*_max_ = 541 nm).

In addition to the UV-visible absorption spectra, the highest occupied molecular orbital (HOMO) and lowest unoccupied molecular orbital (LUMO) levels of photosensitizing dyes play important roles in TiO_2_-based photocatalysts and DSSCs [12]. Electron injection from the LUMO of the photoexcited dye to the CB of TiO_2_ is the first process in TiO_2_-based photocatalysis and DSSCs. The HOMO level is related to the reduction of the oxidized dye formed by electron injection. Therefore, FMO calculations of CV using CPCM in EtOH were performed. The FMOs and energy diagrams of CV are shown in Figures S3 and S4.

According to the TD-DFT calculations, the UV-visible absorption spectra of CV are dominated by the transition from the ground state to the singlet excited state attributed to the HOMO–LUMO transition. The HOMO and LUMO of CV are distributed over the whole molecule due to its extended π-conjugation. In the cases of CV_0_ and CV_iso_, the LUMOs, in particular, are delocalized around the aromatic rings with the imino moiety. This result suggests electron transfer from the aromatic ring with the amino group to the region near the imino moiety, i.e., the amino group acts as a donor. The LUMO levels of CV_0_ and CV_iso_ were calculated to be −2.55 eV and −2.42 eV, respectively. These values are much higher than the CB level of TiO_2_ (−4.0 eV) [10,11,12,27], which indicates the possibility of electron injection from the LUMO of CV_0_ and/or CV_iso_ to the CB of TiO_2_. Additionally, the HOMO levels of CV_0_ and CV_iso_ were calculated to be −5.14 eV and −5.33 eV, respectively. These values are lower than the redox potential of the iodide ion/triiodide ion (I^−^/I_3_^−^, −4.8 eV), which is a common redox couple in the electrolyte solution in DSSCs [10,11,12,27]. This result indicates that the oxidized CV_0_ and CV_iso_ formed by electron injection can be regenerated in the electrolyte. In conclusion, CV was confirmed to act as a photosensitizing dye in TiO_2_-based photocatalysts and DSSCs.

The CV^+^ results calculated at the B3LYP/6-31+G** level (HOMO and LUMO levels of −6.23 eV and −3.67 eV, respectively) suggested the possibility of electron injection and regeneration in the electrolyte. However, the LUMO level calculated at the SVWN/6-31+G** level of theory was lower than the CB level of TiO_2_. These results suggested that the accuracy of both the optical properties and FMO levels of CV^+^ could not be guaranteed. Therefore, CV^+^ was excluded from further study, and only the details of CV_0_ and CV_iso_ were investigated.

### 3.2. Geometry Structures and Characteristics of CV–OTES–C2

**Figure 3:**
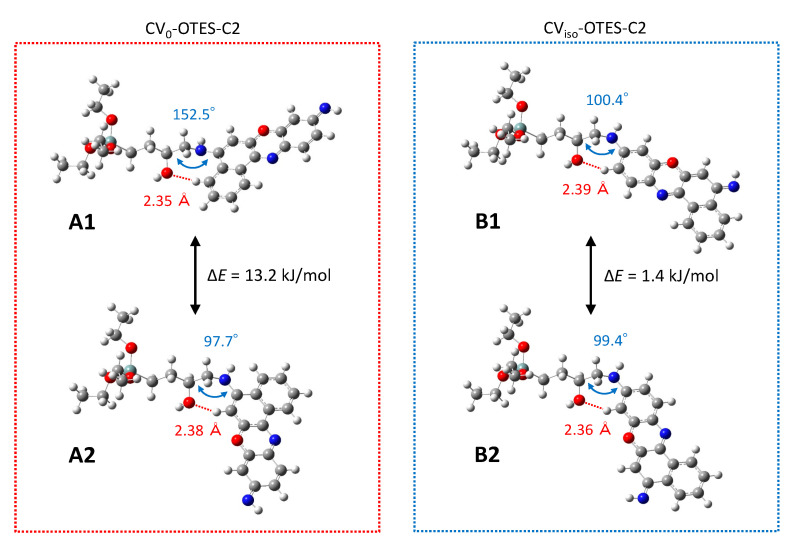
Optimized structures of CV_0_–OTES–C2 and CV_iso_–OTES–C2 in the gas phase, calculated at the B3LYP/6-31G* level of theory. The geometries of CV_0_–OTES–C2 and CV_iso_–OTES–C2 are labeled as A1 and A2 and B1 and B2, respectively. The distance of the CH–O interaction, C–C–N–C dihedral angle and energy difference (Δ*E*) are shown in red, blue and black, respectively (see the text for further details).

Subsequently, geometry optimization of CV_0_ and/or CV_iso_ attached to OTES–C2 in the gas phase was performed (Figure 3). These dyes are denoted as CV_0_–OTES–C2 and CV_iso_–OTES–C2, respectively. Here, it must be assumed that the alkyl alkoxysilane moieties exist in multiple conformations due to C–C bond rotation of the alkyl chain. In this study, the straight alkyl chain configuration was chosen as the starting geometry because the SCAs form a SAMs, attributed to the formation of the siloxane network [13,14].

The hydroxyl group and secondary amine moieties on the alkyl chain resulted from the epoxy-ring-opening reaction between CV_0_ and/or CV_iso_ and OTES–C2. An apparent CH–O interaction between the hydroxyl group of the alkyl chain and the hydrogen atom of the aromatic ring of CV_0_ and/or CV_iso_ can be observed in CV_0_–OTES–C2 and CV_iso_–OTES–C2. The calculated distances between the oxygen and hydrogen atoms were in the range 2.35–2.39 Å, which is shorter than the sum of the van der Waals radii of the oxygen and hydrogen atoms (2.72 Å) [31]. This result further supported the occurrence of the CH–O interaction.

The CH–O interaction was expected to affect the geometry of CV–OTES–C2. In particular, a dihedral angle distortion was expected due to the steric hindrance of the condensed aromatic ring. To confirm this, geometry optimizations of CV_0_–OTES–C2 and CV_iso_–OTES–C2 were performed with different torsional angles for the secondary amine moieties and the dihedral angles of the secondary amine moieties (C–C–N–C dihedral angle, see Figure 3) were examined. The optimized structures of CV_0_–OTES–C2 and CV_iso_–OTES–C2 were labeled as A1/A2 and B1/B2, respectively. As expected, the C–C–N–C dihedral angle of CV_0_–OTES–C2 (conformation A1) was 152.5°, which represented a remarkable distortion compared to that in the other conformations. Since this distortion of the dihedral angle may result in energy differences (sum of electronic and thermal energy; Δ*E*) between the different geometries, frequency analysis was then performed. The Δ*E* between the different optimized geometries of CV_0_–OTES–C2 (conformations A1 and A2) was 13.2 kJ/mol, with a bias toward A2. On the other hand, the two conformations of CV_iso_–OTES–C2 (conformation B1 and B2) were competitive, as Δ*E* was 1.4 kJ/mol. The Δ*E* between the conformations of CV–OTES–C2 was clearly due to the condensed aromatic rings of CV and the CH–O interaction. Based on the above results, it was concluded that the CH–O interaction influences the conformation of CV–OTES–C2.

**Figure 4:**
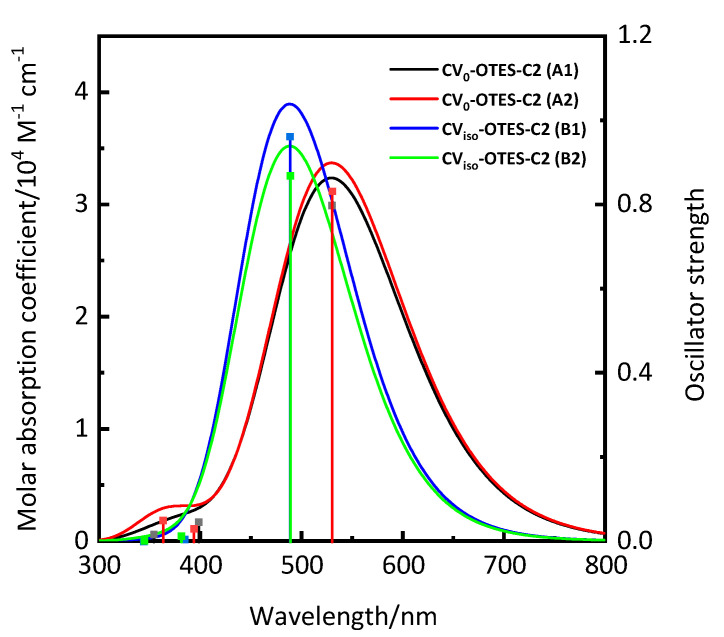
Simulated UV-visible absorption spectra of CV_0_–OTES–C2 and CV_iso_–OTES–C2 using conductor-like polarized continuum model (CPCM) in ethanol (EtOH). Calculations were carried out at the TD-B3LYP/6-31+G* level of theory. Vertical lines indicate the calculated oscillator strengths.

Figure 4 and Table 2 show the simulated UV-visible absorption spectra and calculated optical properties of CV_0_–OTES–C2 and CV_iso_–OTES–C2 using CPCM in EtOH. The *λ*_max_ of CV–OTES–C2 was red-shifted by 12–22 nm compared with that of isolated CV. Additionally, the LHE increased slightly. These results indicated that CV–OTES–C2 was more promising as a photosensitizing dye than isolated CV.

**Figure 5:**
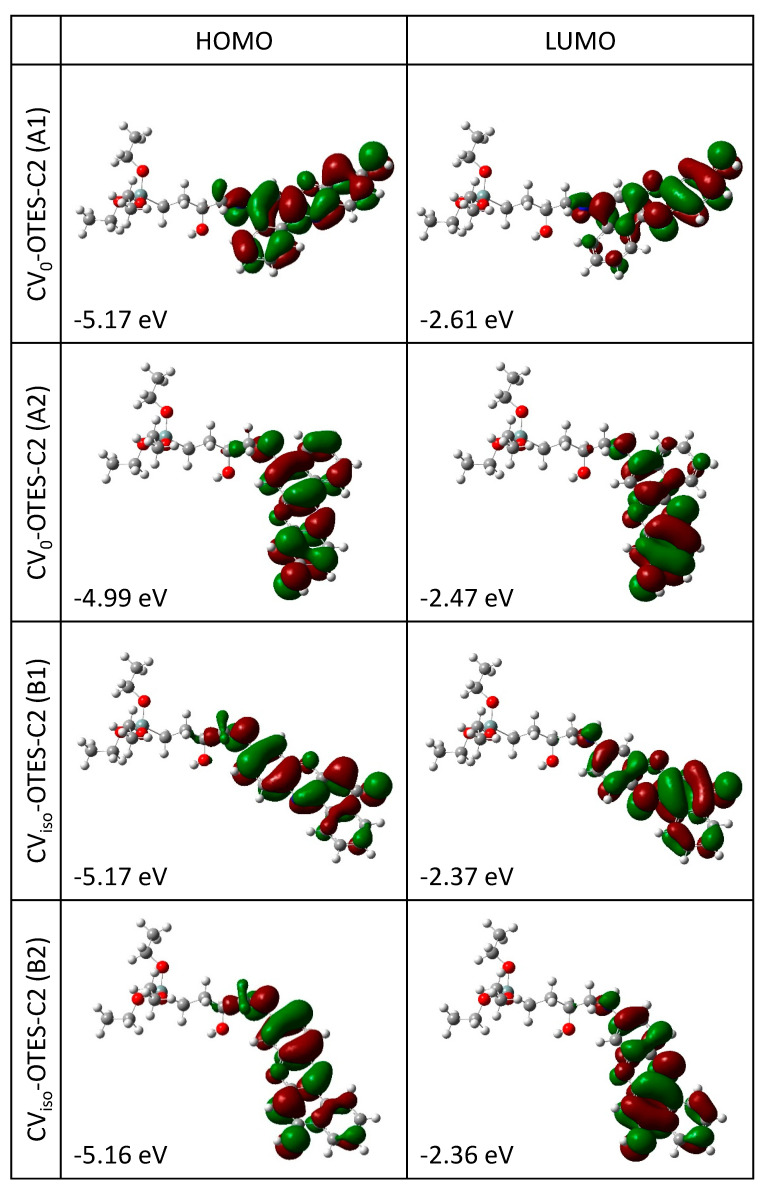
Highest occupied molecular orbitals (HOMO) and lowest unoccupied molecular orbitals (LUMOs) of CV_0_–OTES–C2 and CV_iso_–OTES–C2 using CPCM in EtOH. Calculations were carried out at the B3LYP/6-31G* level of theory. Surface isovalue: 0.02 e/bohr^3^.

**Figure 6:**
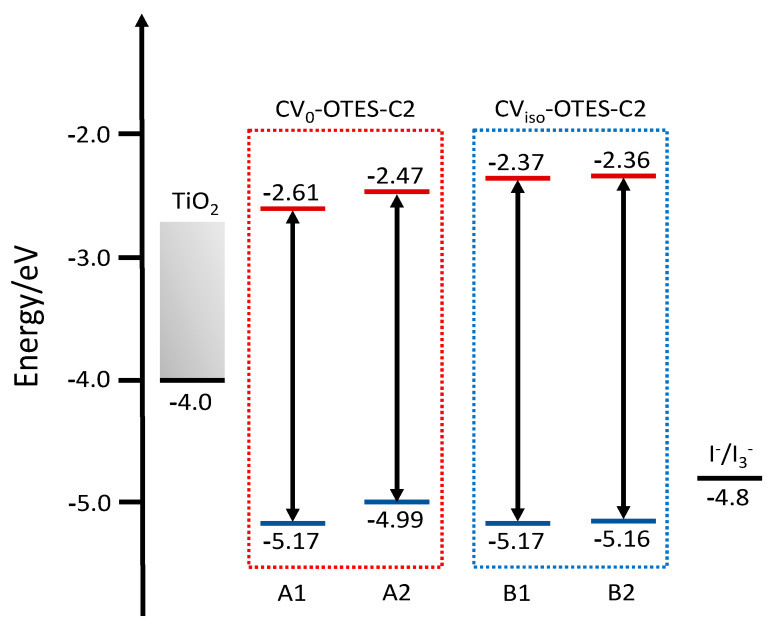
Calculated energy levels of the HOMOs (blue lines) and LUMOs (red lines) for CV_0_–OTES–C2 and CV_iso_–OTES–C2 using CPCM in EtOH.

FMO calculations of CV–OTES–C2 (Figure 5) were performed, and energy diagrams were constructed (Figure 6). The HOMOs of CV–OTES–C2 are distributed even near the secondary amine moieties; the red-shift of *λ*_max_ was attributed to this result. The LUMOs of CV–OTES–C2 are delocalized around the aromatic ring with the imino moiety, similar to isolated CV. The LUMO levels of CV–OTES–C2 were in the range of −2.36 eV to −2.61 eV, which are higher than the CB level of TiO_2_ (−4.0 eV). Additionally, the calculated HOMO levels (−4.99 eV to −5.17 eV) of CV–OTES–C2 are lower than the redox potential of I^−^/I_3_^−^ (−4.8 eV) in an electrolyte solution. These results confirm that CV–OTES–C2 could act as a photosensitizing dye in TiO_2_-based photocatalysts and DSSCs. However, the distribution of the LUMOs around the aromatic ring with the imino moiety may be unfavorable in terms of electron injection, because the LUMOs are located relatively distant from the TiO_2_ surface.

### 3.3. Effect of the Alkyl Chain Length of OTES–Cn

To determine the effect of the alkyl chain length of OTES–Cn, the UV-visible absorption spectra and FMOs of CV_0_–OTES–C4, CV_0_–OTES–C8, CV_iso_–OTES–C4 and CV_iso_–OTES–C8 were investigated. The geometries of these molecules are illustrated in Figure S5. According to frequency analysis, the bent molecular configuration (A2) was dominant for CV_0_–OTES–C2; however, CV_0_–OTES–C2 adsorbed on the TiO_2_ surface may adopt the unbent molecular configuration (A1) due to the large steric hindrance between adjacent CV moieties and/or between the SAM surface and CV moieties (see below for further details). Therefore, the details of the unbent molecular configurations of CV_0_–OTES–Cn and CV_iso_–OTES–Cn (A1 and B1) were investigated. The simulated UV-visible absorption spectra of the dyes did not exhibit significant differences. The alkyl chain length of OTES–Cn did not affect the optical properties of CV–OTES–Cn (Figure S6). It should be noted that the gas-phase spectra were compared because the TD-DFT calculation for CV–OTES–C8 using CPCM in EtOH was very expensive. As would be expected from this result, the distributions of the FMOs and the energy levels of the HOMOs and LUMOs of CV–OTES–Cn were also in good agreement (Figures S7 and S8). Consequently, the alkyl chain length of OTES–Cn was not confirmed to significantly affect the UV-visible absorption spectra and FMOs of CV–OTES–Cn. In other words, if the photocurrent of actual TiO_2_-based photocatalysts and DSSCs is enhanced by the alkyl chain length of OTES–Cn, it may be due to the controlled molecular orientation of CV due to the formation of the SAMs.

### 3.4. Configuration of CV–OTES–C2 Adsorbed on a TiO_2_ Cluster

To understand the structure of CV–OTES–C2 adsorbed on the TiO_2_ surface, geometry optimization of a TiO_2_ cluster and CV_0_–OTES–C2 or CV_iso_–OTES–C2 adsorbed on the TiO_2_ cluster were performed. For the geometry optimization of the TiO_2_ cluster, the anatase TiO_2_ (101) surface model was selected because anatase is known to exhibit higher photocatalytic activity than rutile and the (101) surface has the lowest surface energy [32]. A cluster size of Ti_6_O_15_H_6_ was employed, and the cluster optimized at the B3LYP/6-31G*/LanL2DZ level of theory to compromise between computational costs and accuracy (Figure S9).

**Figure 7:**
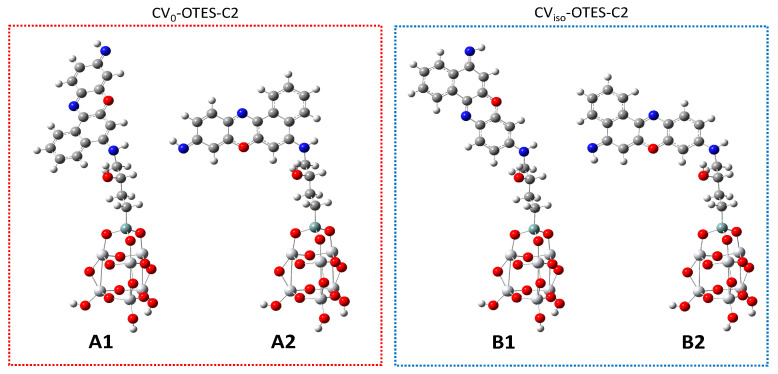
Optimized structures of CV–OTES–C2 adsorbed on the TiO_2_ cluster in the gas phase, calculated using the B3LYP/6-31G*/LanL2DZ level of theory. The conformations of CV_0_–OTES–C2 and CV_iso_–OTES–C2 are labeled as A1 and A2 and B1 and B2, respectively.

Figure 7 illustrates CV–OTES–C2 adsorbed conformations on the TiO_2_ cluster (tridentate bridging model, calculated in the gas phase). The calculated bond lengths between the alkoxysilane oxygen atoms and connected titanium atoms were ~1.86 Å, comparable to the Ti–O bond lengths in the Ti_6_O_15_H_6_ cluster (1.80–1.84 Å). These bond lengths indicated strong chemical adsorption of CV–OTES–C2 onto the TiO_2_ (101) surface.

The molecular length provided information related to the conformation of CV–OTES–C2 adsorbed on the TiO_2_ surface. The long axes (distance between the silane atom on the alkoxysilane moiety and the nitrogen atom on the imino moiety) of CV_0_–OTES–C2 and CV_iso_–OTES–C2 in the unbent conformations (A1 and B1) were 15.9 Å (Figure S10). In comparison, those of the bent conformations (A2 and B2) were 12.1 Å and 12.3 Å, respectively, and had shorter contact distances between the CV moiety and the TiO_2_ surface compared to geometries A1 and B1. Additionally, the short axes (the distance between the nitrogen atoms on the imino moieties and those on the secondary amine moieties) of the bent conformations were 9.5 Å and 9.6 Å, respectively. If a SAMs based on OTES–Cn was present on the TiO_2_ surface, the bent molecular geometry would be unlikely due to the steric hindrance between adjacent CV moieties and/or between the SAMs surface and CV moieties. Therefore, it was expected that the unbent conformation of CV–OTES–C2 would predominate on the TiO_2_ surface.

### 3.5. FMO, DOS Spectra and MEP of CV–OTES–C2 Adsorbed on the TiO_2_ Cluster

**Figure 8:**
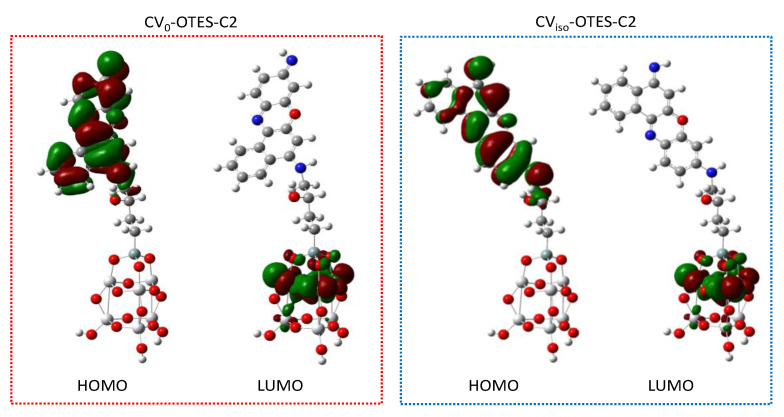
HOMOs and LUMOs of CV_0_–OTES–C2 or CV_iso_–OTES–C2 adsorbed on the TiO_2_ cluster using CPCM in EtOH, calculated at the B3LYP/6-31G*/LanL2DZ level of theory. Surface isovalue: 0.02 e/bohr^3^.

**Figure 9:**
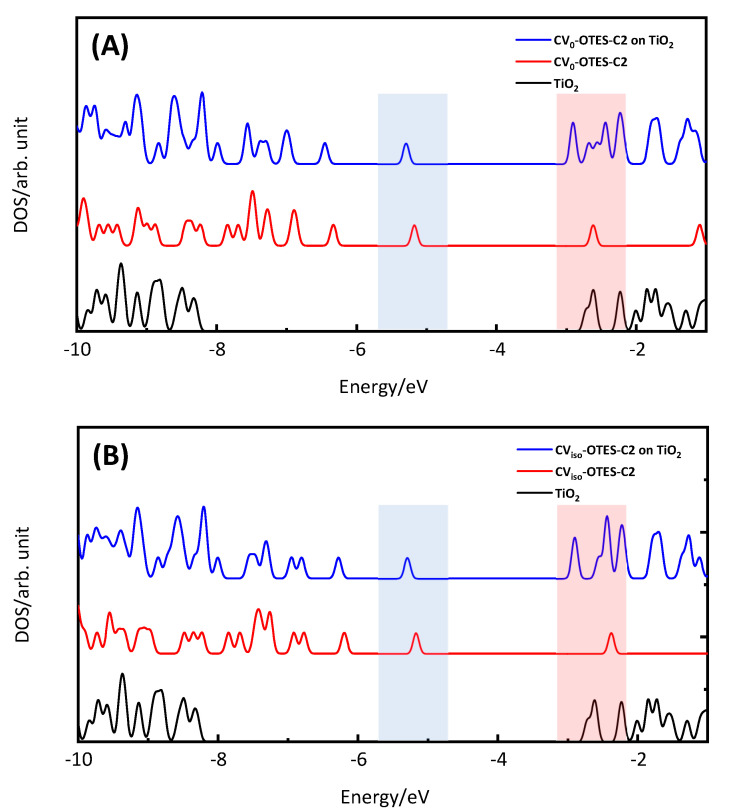
Density of state (DOS) spectra of (**A**) CV_0_–OTES–C2 and (**B**) CV_iso_–OTES–C2 adsorbed on the TiO_2_ cluster using CPCM in EtOH. Black, red and blue lines correspond to the TiO_2_ cluster, isolated CV–OTES–C2 and CV–OTES–C2 adsorbed on the TiO_2_ cluster. Blue and red bars indicate the HOMO and LUMO.

Subsequently, the FMOs and DOS spectra of CV–OTES–C2 in the unbent conformations (A1 and B1) adsorbed on the TiO_2_ cluster were calculated to investigate the electron injection from CV–OTES–C2 to TiO_2_ (Figure 8 and Figure 9). As in the case of isolated CV–OTES–C2, the HOMOs of CV–OTES–C2 adsorbed on the TiO_2_ cluster were distributed around the CV and secondary amine moieties. At the same time, the LUMOs were located around the titanium atoms connected to the alkoxysilane oxygens. This result indicates that electron injection would occur from CV–OTES–C2 to TiO_2_. However, it should be noted that the CB level of Ti_6_O_15_H_4_ was estimated to be −2.72 eV by the FMO calculation using CPCM in EtOH.

According to the DOS spectra, the HOMO and LUMO levels of CV–OTES–C2 were downshifted by adsorption onto TiO_2_. In particular, the downshift of the LUMO level was remarkable and suggested degeneracy between the CB level of TiO_2_ and the LUMO level of CV–OTES–C2. This bandgap reduction may induce efficient visible light absorption and electron injection.

**Figure 10:**
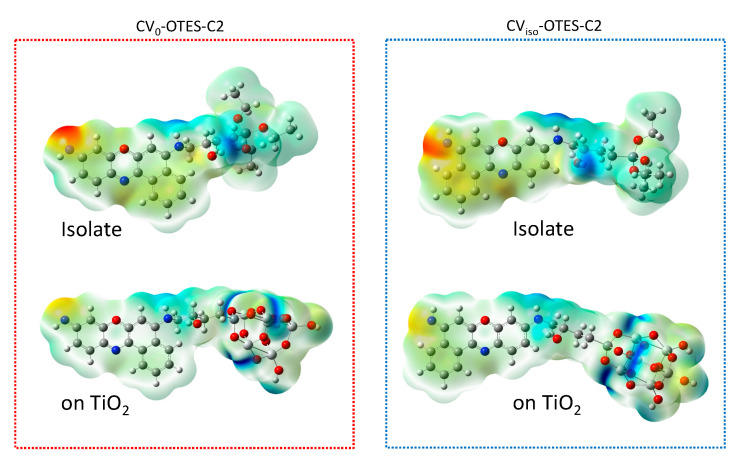
Molecular electrostatic potential (MEP) of isolated CV–OTES–C2 and CV_iso_–OTES–C2 adsorbed on the TiO_2_ cluster using CPCM in EtOH. Red and blue indicate electron-rich (negative charge) and electron-deficient regions (positive charge), respectively; the electrostatic potential increases in the order red < orange < yellow < green < cyan < blue. Color code of the MEP maps of the isolated CV–OTES–C2 and the CV–OTES–C2 adsorbed on the TiO_2_ cluster ranged from −0.078 a.u. to 0.078 a.u. and from −0.11 a.u. to 0.11 a.u., respectively.

The MEPs of isolated CV–OTES–C2 and CV–OTES–C2 adsorbed on the TiO_2_ cluster were calculated to complement the FMO calculations and DOS spectra (Figure 10). The MEP identifies the nucleophilic reaction sites and electrophilic reaction sites [27]. In all dyes, the imino moiety exhibited the highest nucleophilic potential. In isolated CV–OTES–C2, the hydrogen atom of the hydroxyl group and the secondary amine moiety on the alkyl chain exhibited electrophilic potentials. On the other hand, for CV–OTES–C2 to be adsorbed on the TiO_2_ cluster, the titanium atoms connected to the alkoxysilane oxygens needed to have the greatest electrophilic potential. This indicates that the electron injection to TiO_2_ occurs via the alkoxysilane moiety, i.e., these results support those of the FMO calculations and DOS spectra.

### 3.6. Simulated Short-Circuit Current Density

The PCE of DSSC is calculated using the following equation [10,27]:

PCE = (*J*_sc_ × *V*_oc_ × FF)/*I*_0_ (2)

Here, *J*_sc_, *V*_oc_, FF and *I*_0_ are the short-circuit current density, open-circuit voltage, fill factor and incident light flux, respectively. *J*_sc_ and *V*_oc_ are important factors in the enhancement of the PCE. *J*_sc_ is defined by the following equation [10,11,27]:

*J*_sc_ = *∫* (LHE(*λ*) × *Φ*_inject_ × *η*_collect_) *dλ* (3)

where LHE(*λ*), *Φ*_inject_ and *η*_collect_ are the LHE at a given wavelength, the electron injection efficiency and the charge collection efficiency, respectively. It was assumed that all the components of the DSSC were constant except for the photosensitizing dye. Hence, *η*_collect_ can be assumed to be constant. *Φ*_inject_ is related to the injection driving force (∆*G*_inject_) of the electrons injected from the excited dyes to the TiO_2_. ∆*G*_inject_ can be estimated using the following equation [10,11,27]:

∆*G*_inject_ = *E*^dye*^ − *E*_CB_ = (*E*^dye^ − *E*_S1_) − *E*_CB_ (4)

*E*^dye*^, *E*^dye^, *E*_CB_ and *E*_S1_ are the oxidation potential of the photosensitizing dye in the excited state, the redox potential of the photosensitizing dye in the ground state, the reduction potential of the CB level of TiO_2_ (4.0 eV) and the energy of the singlet excited state. *E*^dye^ can be estimated as the negative of the HOMO level [11]. *E*_S1_ is the vertical transition energy associated with the *λ*_max_ value obtained from the UV-visible absorption spectra and is listed in Table 1 and Table 2. The *E*^dye^, *E*^dye*^ and ∆*G*_inject_ values of isolated CV–OTES–C2 are listed in Table 3.

The ∆*G*_inject_ values for the A1, A2, B1 and B2 conformations were −1.17 eV, −1.35 eV, −1.36 eV and −1.38 eV; these values were more negative than that of *E*_CB_. This result indicates that the excited state of CV–OTES–C2 lies above the CB edge of TiO_2_, i.e., that electron injection should occur from the excited CV–OTES–C2 to the CB of TiO_2_.

The ∆*G*_inject_ of CV_0_–OTES–C2 (A1) is the lowest, suggesting that conformation A1 would exhibit slower electron injection and a lower *J*_SC_ than the other geometries. However, the bent molecular conformations of CV–OTES–C2 (A2 and B2) may not form on the TiO_2_ surface (see above for further details). The UV-visible absorption spectrum of conformation A1 also has a broader absorption band than geometry B1 (Figure 4). The LHE depends not only on absorbance but also on the width and tails of the absorption band. Photosensitizing dyes with wider absorption bands in the visible/IR region can achieve much higher LHEs [11]. Therefore, it is expected that the characteristics of CV_0_–OTES–C2 (A1) would contribute significantly to enhancing the photocurrent in TiO_2_-based photocatalysts and DSSCs.

## 4. Conclusions

This article reported a DFT and TD-DFT study of CV–OTES–Cn to determine the influence of OTES–Cn on photosensitizing dyes’ geometry. The potential of CV–OTES–Cn to act as a photosensitizing dye was also evaluated. The hydroxyl group formed by the epoxy-opening reaction resulted in a CH–O interaction between CV and the covalently attached OTES–Cn. This CH–O interaction significantly influenced the conformation of CV–OTES–Cn and distorted the C–C–N–C dihedral angle due to the steric hindrance of the condensed aromatic ring of CV. According to TD-DFT calculations (simulated UV-visible absorption spectra), CV–OTES–Cn has excellent optical properties and should act as a promising photosensitizer for TiO_2_-based photocatalysts and DSSCs. Additionally, the results of FMO calculations, DOS spectra and MEP calculations indicated that electron injection proceeds from CV–OTES–Cn to TiO_2_. In particular, the characteristics of the unbent molecular geometry of CV–OTES–Cn (conformation A1) should contribute significantly to enhancing photocurrent in TiO_2_-based photocatalysts and DSSCs. Overall, the following conclusions were drawn: (i) SAMs based on the OTES–Cn on the TiO_2_ surface can control CV conformation covalently attached to the terminal functional group of OTES–Cn (CV–OTES–Cn); (ii) CV–OTES–Cn has potential as a promising photosensitizer for TiO_2_-based photocatalysts and DSSCs. This article’s findings should enhance covalent attachment strategies between photosensitizing dyes and TiO_2_ and improve TiO_2_-based photocatalysts and DSSCs.

## Figures and Tables

**Table 1:** Calculated maximum absorption peaks (*λ*_max_), molar absorption coefficients (*ε*), energies of singlet excited states (*E*_S1_), oscillator strengths (*f*) and light-harvesting efficiencies (LHE) of CV_0_, CV_iso_ and CV^+^.

Dyes	Conditions	*λ*_max_ (nm)	*ε* (10^4^ M^−1^ cm^−1^)	*E*_S1_ (eV)	*f*	LHE
CV_0_	gas phase	491	2.09	2.52	0.513	0.69
	in EtOH	518	3.10	2.39	0.7643	0.83
CV_iso_	gas phase	432	2.44	2.87	0.5968	0.75
	in EtOH	467	3.25	2.66	0.8008	0.84
CV^+^	in EtOH	510	3.51	2.43	0.8641	0.86
CV^+ a^	in EtOH	541	2.76	2.29	0.6637	0.78

a: calculation method was using SVWN/6-31+G** level.

**Table 2:** Calculated maximum absorption peaks (*λ*_max_), molar absorption coefficients (*ε*), energies of singlet excited states (*E*_S1_), oscillator strengths (*f*) and light-harvesting efficiencies (LHE) of CV_0_–OTES–C2 and CV_iso_–OTES–C2.

Dyes	*λ*_max_ (nm)	*ε* (10^4^ M^−1^ cm^−1^)	*E*_S1_ (eV)	*f*	LHE
CV_0_–OTES–C2 (A1)	530	3.24	2.34	0.7979	0.84
CV_0_–OTES–C2 (A2)	530	3.37	2.34	0.8317	0.85
CV_iso_–OTES–C2 (B1)	488	3.89	2.53	0.9614	0.89
CV_iso_–OTES–C2 (B2)	489	3.52	2.54	0.8688	0.86

**Table 3:** Redox potential in the ground state (*E*^dye^), oxidation potential in the excited state (*E*^dye*^) and injection driving force (∆*G*_inject_) of isolated CV–OTES–C2.

Dyes	*E*^dye^ (eV)	*E*^dye*^ (eV)	∆*G*_inject_ (eV)
CV_0_–OTES–C2 (A1)	5.17	2.83	−1.17
CV_0_–OTES–C2 (A2)	4.99	2.65	−1.35
CV_iso_–OTES–C2 (B1)	5.17	2.64	−1.36
CV_iso_–OTES–C2 (B2)	5.16	2.62	−1.38

## Supplementary Materials

The following are available online at https://www.mdpi.com/2079-4991/10/10/1958/s1, Figure S1: Chemical structures of 4-(triethoxysilyl)butane-1,2-epoxide (OTES–C2), 4-oxiran-2-ylbutyl-triethoxysilane (OTES–C4) and 8-oxiran-2-yloctyltriethoxysilane (OTES–C8); Figure S2: Optimized structures of CV_0_, CV_iso_ and CV^+^ in the gas phase. Geometry optimization was performed at the B3LYP/6-31G* and SVWN/6-31+G** levels of theory. (See Section 2 and Section 3 for further details); Figure S3: HOMO and LUMO of CV using CPCM in EtOH. In the cases of CV_0_ and CV_iso_, the B3LYP/6-31G* level of theory was employed for the FMO calculations. The HOMO and LUMO of CV^+^ were calculated using the B3LYP/6-31+G** and SVWN/6-31+G** levels of theory. Surface isovalue: 0.02 e/bohr^3^; Figure S4: Calculated energy levels of the HOMOs (blue lines) and the LUMOs (red lines) of CV_0_, CV_iso_ and CV^+^ using CPCM in EtOH; Figure S5: Optimized structures of CV_0_–OTES–C4, CV_0_–OTES–C8, CV_iso_–OTES–C4 and CV_iso_–OTES–C8 in the gas phase, calculated at the B3LYP/6-31G* level of theory; Figure S6: Simulated UV-visible absorption spectra of (A) CV_0_–OTES–Cn and (B) CV_iso_–OTES–Cn in the gas phase, calculated using the TD-B3LYP/6-31+G* level of theory. Vertical lines indicate the calculated oscillator strength; Figure S7: HOMOs and LUMOs of CV–OTES–C4 and CV–OTES–C8 using CPCM in EtOH, calculated using the B3LYP/6-31G* level of theory. Surface isovalue: 0.02 e/bohr^3^; Figure S8: Calculated energy levels of the HOMOs (blue lines) and LUMOs (red lines) for CV–OTES–Cn using CPCM in EtOH; Figure S9: Optimized structure of a Ti_6_O_15_H_6_ cluster modeling the anatase (101) surface, calculated using the B3LYP/6-31G*/LanL2DZ level of theory. Gray, red and white atoms correspond to Ti, O and H, respectively, Figure S10: Molecular length of CV–OTES–C2 adsorbed on the TiO_2_ cluster.

## References

[1] Lu G., Liu X., Zhao L., Zhang P., Gao Y. (2020). Synergistic photocatalytic performance of chemically modified amino phthalocyanine-GPTMS/TiO_2_ for the degradation of Acid Black 1. Inorg. Chem. Commun..

[2] Kakiage K., Aoyama Y., Yano T., Oya K., Fujisawa J.-I., Hanaya M. (2015). Highly-efficient dye-sensitized solar cells with collaborative sensitization by silyl-anchor and carboxy-anchor dyes. Chem. Commun..

[3] Bhat V.T., Duspara P.A., Seo S., Abu Bakar N.S.B., Greaney M.F. (2015). Visible light promoted thiol-ene reactions using titanium dioxide. Chem. Commun..

[4] Rodriguez-Gonzalez V., Obregon S., Patron-Soberano O.A., Terashima C., Fujishima A. (2020). An approach to the photocatalytic mechanism in the TiO_2_-nanomaterials microorganism interface for the control of infectious processes. Appl. Catal. B Environ..

[5] Paszkiewicz-Gawron M., Makurat S., Rak J., Zdrowowicz M., Lisowski W., Zaleska-Medynska A., Kowalska E., Mazierski P., Luczak J. (2020). Theoretical and experimental studies on the visible light activity of TiO_2_ modified with halide-based ionic liquids. Catalysts.

[6] Rahimi R., Moghaddas M.M., Zargari S. (2013). Investigation of the anchoring silane coupling reagent effect in porphyrin sensitized mesoporous V-TiO_2_ on the photodegradation efficiency of methyl orange under visible light irradiation. J. Sol-Gel Sci. Technol..

[7] Sil M.C., Kavungathodi M.F.M., Nithyanandhan J. (2019). Effect and position of spiro-bipropylenedioxythiophene π-spacer in donor-π-spacer-acceptor dyes for dye-sensitized solar cell. Dyes Pigment..

[8] Zhu H.-C., Zhang J., Wang Y.-L. (2018). Adsorption orientation effects of porphyrin dyes on the performance of DSSC: Comparison of benzoic acid and tropolone anchoring groups binding onto the TiO_2_ anatase (101) surface. Appl. Surf. Sci..

[9] Ooyama Y., Shimada Y., Inoue S., Nagano T., Fujikawa Y., Komaguchi K., Imae I., Harima Y. (2011). New molecular design of donor-π-acceptor dyes for dye-sensitized solar cells: Control of molecular orientation and arrangement on TiO_2_ surface. New J. Chem..

[10] Yang Z., Liu C., Shao C., Lin C., Liu Y. (2015). First-Principles Screening and Design of Novel Triphenylamine-Based D-π-A Organic Dyes for Highly Efficient Dye-Sensitized Solar Cells. J. Phys. Chem. C.

[11] Ud-Din Khan S., Mahmood A., Rana U.A., Haider S. (2015). Utilization of electron-deficient thiadiazole derivatives as π-spacer for the red shifting of absorption maxima of diarylamine-fluorene based dyes. Theor. Chem. Acc..

[12] Li P., Wang Z., Zhang H. (2019). Rigidified and expanded N-annulated perylenes as efficient donors in organic sensitizers for application in solar cells. Phys. Chem. Chem. Phys..

[13] Zhan F., Xiong L., Liu F., Li C. (2019). Grafting hyperbranched polymers onto TiO_2_ nanoparticles via thiol-yne click chemistry and its effect on the mechanical, thermal and surface properties of polyurethane coating. Materials.

[14] Meroni D., Lo Presti L., Di Liberto G., Ceotto M., Acres R.G., Prince K.C., Bellani R., Soliveri G., Ardizzone S. (2017). A Close Look at the Structure of the TiO_2_-APTES Interface in Hybrid Nanomaterials and Its Degradation Pathway: An Experimental and Theoretical Study. J. Phys. Chem. C.

[15] Krishnakumar B., Balakrishna A., Arranja C.T., Dias C.M.F., Sobral A.J. (2017). Chemically modified amino porphyrin/TiO_2_ for the degradation of Acid Black 1 under day light illumination. Spectrochim. Acta Part A Mol. Biomol. Spectrosc..

[16] Luitel T., Zamborini F.P. (2013). Covalent Modification of Photoanodes for Stable Dye-Sensitized Solar Cells. Langmuir.

[17] Pandit B., Luitel T., Cummins D.R., Thapa A.K., Druffel T., Zamborini F., Liu J. (2013). Spectroscopic Investigation of Photoinduced Charge-Transfer Processes in FTO/TiO_2_/N719 Photoanodes with and without Covalent Attachment through Silane-Based Linkers. J. Phys. Chem. A.

[18] Abdulelah H., Ali B., Mahdi M.A., Abdullah A.Q., Hassan J.J., Al-Taay H.F., Jennings P. (2016). Fabrication and characterization of porous CdS/dye sensitized solar cells. J. Sol. Energy.

[19] Tan H., Pan C., Wang G., Wu Y., Zhang Y., Yu G., Zhang M. (2014). A comparative study on properties of two phenoxazine-based dyes for dye-sensitized solar cells. Dye. Pigment..

[20] Hong Y., Iqbal Z., Yin X., Cao D. (2014). Synthesis of double D-A branched organic dyes employing indole and phenoxazine as donors for efficient DSSCs. Tetrahedron.

[21] O’Boyle N.M., Tenderholt A.L., Langner K.M. (2008). cclib: A library for package-independent computational chemistry algorithms. J. Comput. Chem..

[22] Hay P.J., Wadt W.R. (1985). Ab initio effective core potentials for molecular calculations. Potentials for potassium to gold including the outermost core orbitals. J. Chem. Phys..

[23] Batsanov S.S. (2001). Van der Waals radii of elements. Inorg. Mater. (Transl. Neorg. Mater.).

[24] Alvarez S. (2013). A cartography of the van der Waals territories. Dalton Trans..

[25] Pinzaru S.C., Falamas A., Dehelean C., Morari C., Venter M. (2013). Double amino functionalized Ag nanoparticles as SERS tags in Raman diagnostic. Croat. Chem. Acta.

[26] Gong Q., Shi W., Li L., Ma H. (2016). Leucine aminopeptidase may contribute to the intrinsic resistance of cancer cells toward cisplatin as revealed by an ultrasensitive fluorescent probe. Chem. Sci..

[27] Al Mamunur Rashid M., Hayati D., Kwak K., Hong J. (2020). Theoretical investigation of azobenzene-based photochromic dyes for dye-sensitized solar cells. Nanomaterials.

[28] Jafari A., Ghanadzadeh A., Tajalli H., Yeganeh M., Moghadam M. (2007). Electronic absorption spectra of cresyl violet acetate in anisotropic and isotropic solvents. Spectrochim. Acta Part A Mol. Biomol. Spectrosc..

[29] Banik S., Hussain S.A., Bhattacharjee D. (2018). Modified aggregation pattern of cresyl violet acetate adsorbed on nano clay mineral layers in Langmuir Blodgett film. J. Photochem. Photobiol. A Chem..

[30] Ostrowski P.P., Fairn G.D., Grinstein S., Johnson D.E. (2016). Cresyl violet: A superior fluorescent lysosomal marker. Traffic.

[31] Itoh Y., Nakashima Y., Tsukamoto S., Kurohara T., Suzuki M., Sakae Y., Oda M., Okamoto Y., Suzuki T. (2019). N^+^-C-H···O Hydrogen bonds in protein-ligand complexes. Sci. Rep..

[32] Oprea C.I., Girtu M.A. (2019). Structure and electronic properties of TiO_2_ nanoclusters and dye-nanocluster systems appropriate to model hybrid photovoltaic or photocatalytic applications. Nanomaterials.

