# Supplementary material for: Computational Study of Cresyl Violet Covalently Attached to the Silane Coupling Agents: Application to TiO2-Based Photocatalysts and Dye-Sensitized Solar Cells
Source: Nanomaterials (Basel). 2020 Oct 1;10(10):1958. doi: 10.3390/nano10101958 (PMC7600687; doi:10.3390/nano10101958)
Supplement: Supplementary file 1 [file nanomaterials-10-01958-s001.pdf]

Supplementary Materials

Computational Study of Cresyl Violet Covalently Attached to the Silane Coupling Agents: Application to TiO_2-based Photocatalysts and Dye-sensitized Solar Cells

Tatsuya Takeshita

Department of Applied Chemistry and Food Science, Fukui University of Technology, 3-6-1 Gakuen, Fukui 910-8505, Japan; takeshita@fukui-ut.ac.jp

Contents

1. Chemical structures of OTES–Cn
2. Optimized structures of CV_0, CV_{iso}, and CV^+ in the gas phase
3. Frontier molecular orbitals of CV using CPCM in EtOH
4. Energy diagram of CV
5. Optimized structures of CV–OTES–C4 and CV–OTES–C8
6. Simulated UV-visible absorption spectra of CV–OTES–Cn
7. Frontier molecular orbitals of CV–OTES–C4 and CV–OTES–C8
8. Energy diagram of CV–OTES–Cn
9. Optimized structure of the TiO_2 cluster
10. Molecular length of CV–OTES–C2 adsorbed on the TiO_2 cluster

---

1. Chemical structures of OTES–Cn

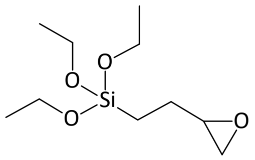

OTES-C2

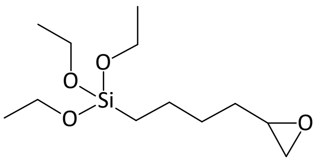

OTES-C4

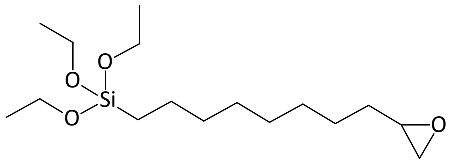

OTES-C8

Figure S1. Chemical structures of 4-(triethoxysilyl)butane-1,2-epoxide (OTES–C2), 4-oxiran-2-ylbutyl-triethoxysilane (OTES–C4), and 8-oxiran-2-yloctyltriethoxysilane (OTES–C8).

2. Optimized structures of CV_0, CV_{iso}, and CV^+ in the gas phase

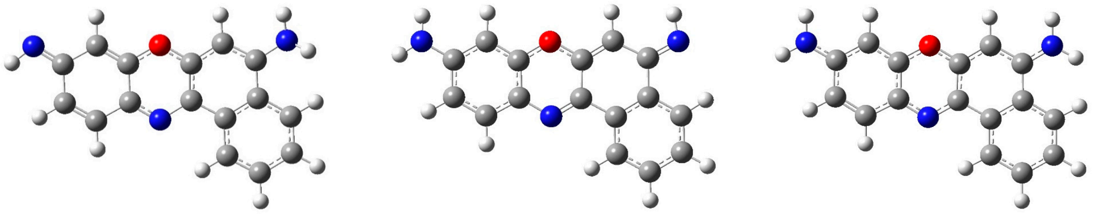

CV_0 CV_{iso} CV^+

Figure S2. Optimized structures of CV_0, CV_{iso}, and CV^+ in the gas phase. Geometry optimization was performed at the B3LYP/6-31G* and/or SVWN/6-31+G** levels of theory. (See Sections 2 and 3 for further details).

3. Frontier molecular orbitals of CV using CPCM in EtOH

	HOMO	LUMO
CV_0	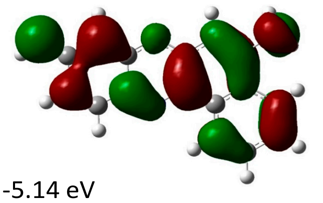 -5.14 eV	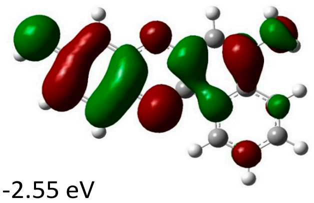 -2.55 eV
CV_{iso}	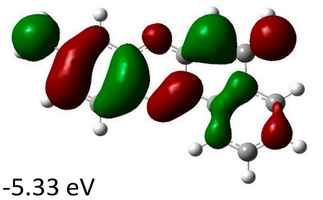 -5.33 eV	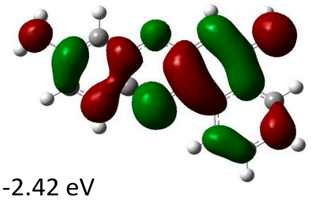 -2.42 eV
CV^+ (B3LYP)	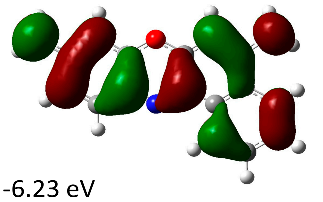 -6.23 eV	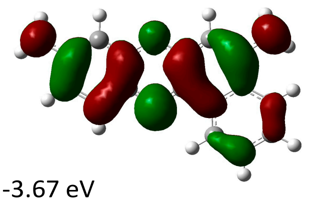 -3.67 eV
CV^+ (SVWN)	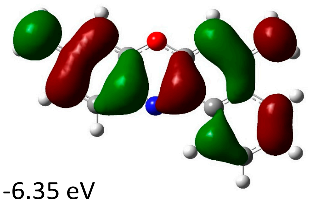 -6.35 eV	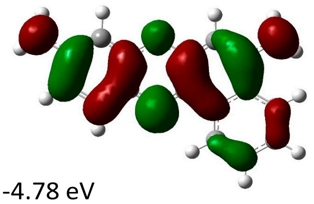 -4.78 eV

Figure S3. HOMO and LUMO of CV using CPCM in EtOH. In the cases of CV_0 and CV_{iso}, the B3LYP/6-31G* level of theory was employed for the FMO calculations. The HOMO and LUMO of CV^+ were calculated using the B3LYP/6-31+G** and/or SVWN/6-31+G** levels of theory. Surface isovalue: 0.02 e/bohr^3.

4. Energy diagram of CV

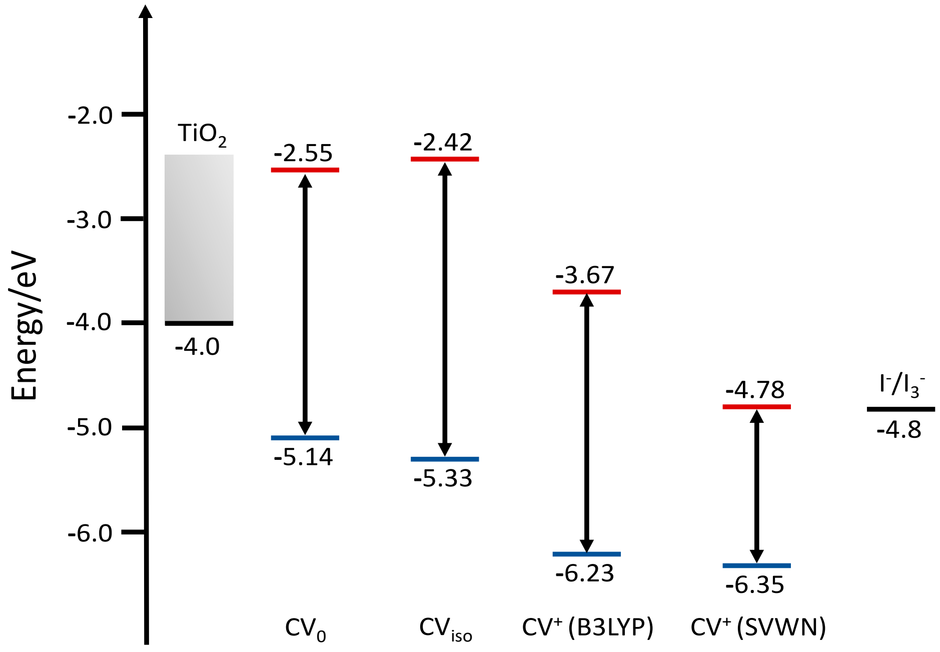

Figure S4. Calculated energy levels of the HOMOs (blue lines) and the LUMOs (red lines) of CV_0, CV_{iso}, and CV^+ using CPCM in EtOH.

5. Optimized structures of CV–OTES–C4 and CV–OTES–C8

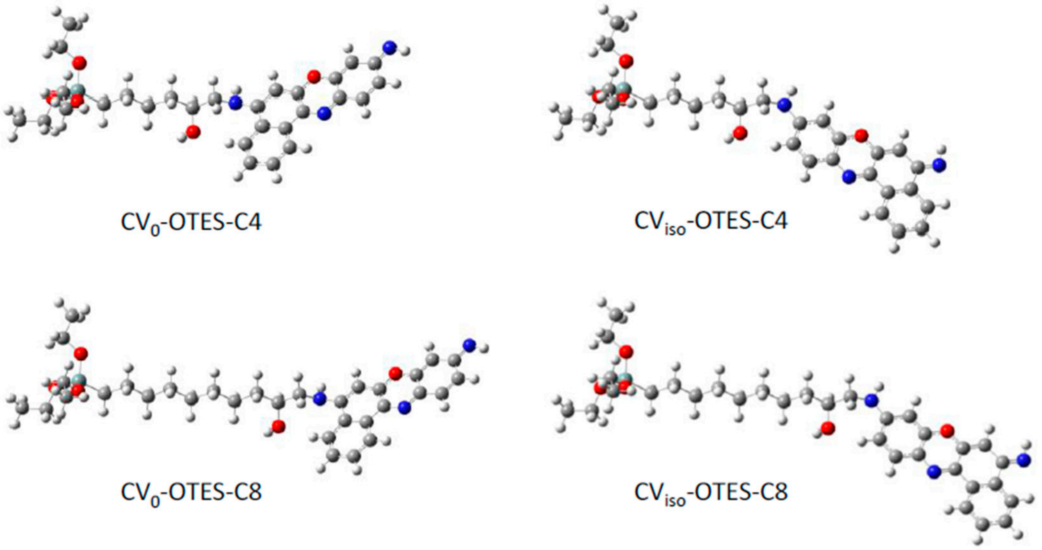

Figure S5. Optimized structures of CV_0–OTES–C4, CV_0–OTES–C8, CV_{iso}–OTES–C4, and CV_{iso}–OTES–C8 in the gas phase, calculated at the B3LYP/6-31G* level of theory.

6. Simulated UV-visible absorption spectra of CV–OTES–Cn

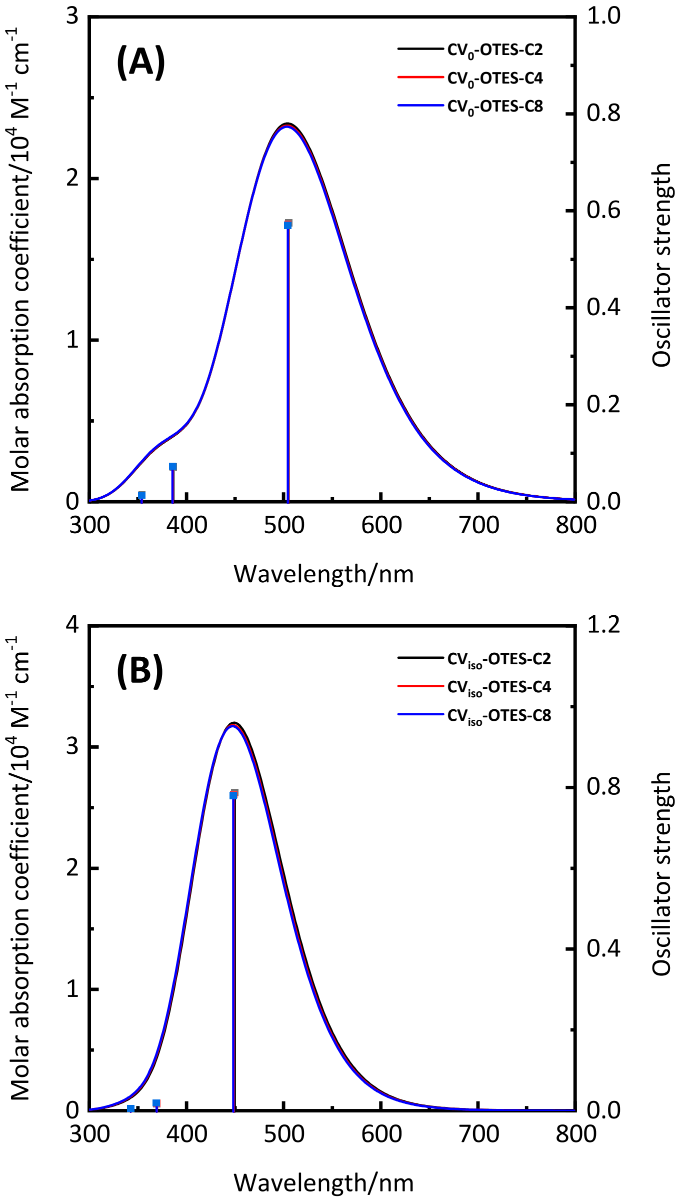

Figure S6. Simulated UV-visible absorption spectra of (**A**) CV_0–OTES–Cn and (**B**) CV_{iso}–OTES–Cn in the gas phase, calculated using the TD-B3LYP/6-31+G* level of theory. Vertical lines indicate the calculated oscillator strength.

7. Frontier molecular orbitals of CV–OTES–C4 and CV–OTES–C8

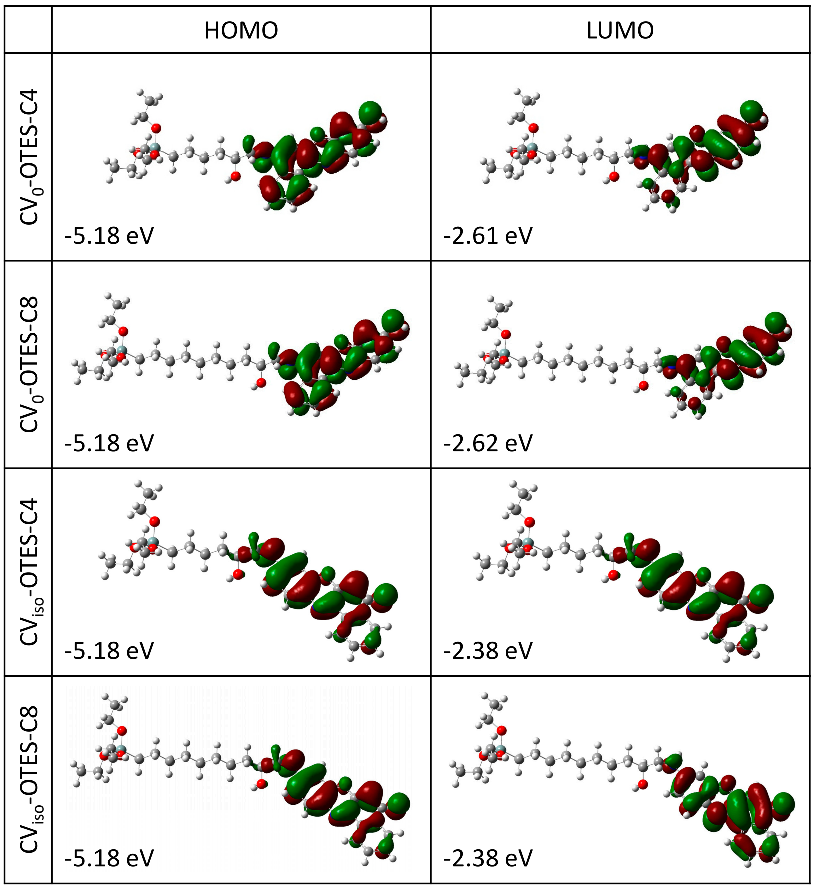

Figure S7. HOMOs and LUMOs of CV–OTES–C4 and CV–OTES–C8 using CPCM in EtOH, calculated using the B3LYP/6-31G* level of theory. Surface isovalue: 0.02 e/bohr³.

8. Energy diagram of CV–OTES–Cn

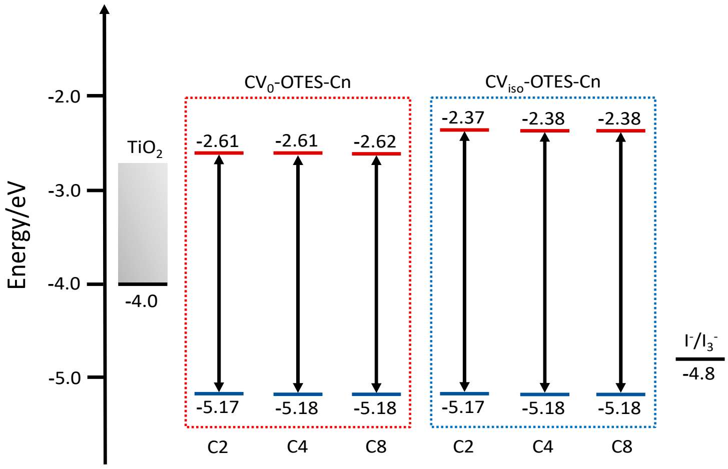

Figure S8. Calculated energy levels of the HOMOs (blue lines) and LUMOs (red lines) for CV–OTES–Cn using CPCM in EtOH.

9. Optimized structure of the TiO_2 cluster

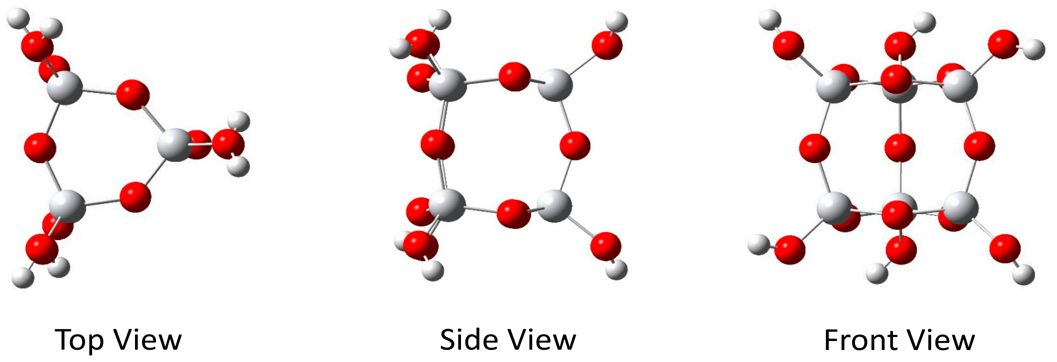

Figure S9. Optimized structure of a $Ti_6O_{15}H_6$ cluster modeling the anatase (101) surface, calculated using the B3LYP/6-31G*/LanL2DZ level of theory. Gray, red, and white atoms correspond to Ti, O, and H, respectively.

10. Molecular length of CV–OTES–C2 adsorbed on the TiO_2 cluster

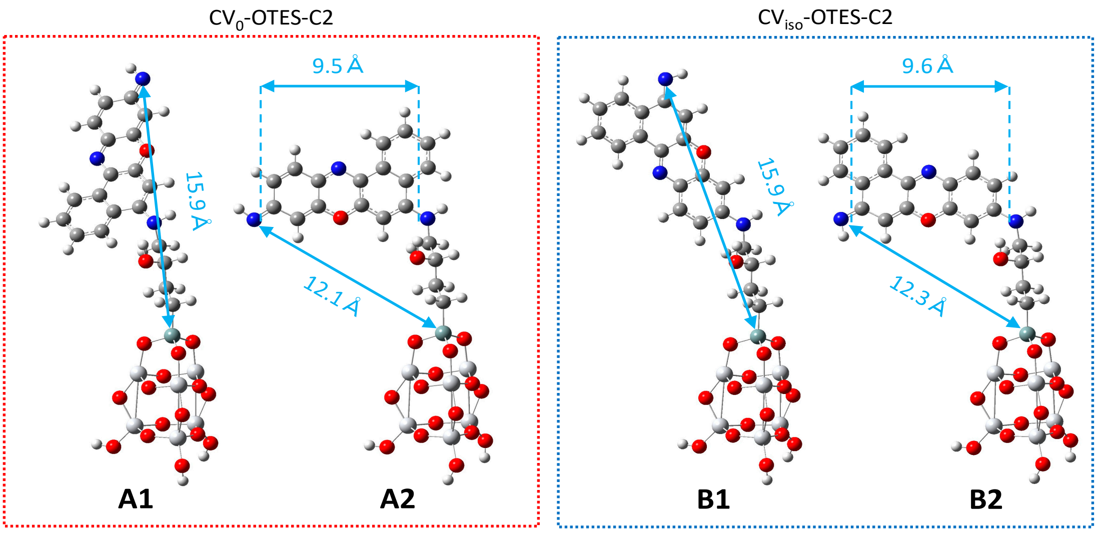

Figure S10. Molecular length of CV–OTES–C2 adsorbed on the TiO_2 cluster.
